# A Plasmonic Spanner for Metal Particle Manipulation

**DOI:** 10.1038/srep15446

**Published:** 2015-10-20

**Authors:** Yuquan Zhang, Wei Shi, Zhe Shen, Zhongsheng Man, Changjun Min, Junfeng Shen, Siwei Zhu, H. Paul Urbach, Xiaocong Yuan

**Affiliations:** 1 Institute of Micro and Nano Optics, Key Laboratory of Optoelectronic Devices and Systems of Ministry of Education and Guangdong Province, College of Optoelectronic Engineering, Shenzhen University, Shenzhen, 518060, China; 2 Institute of Modern Optics, Nankai University, Tianjin, 300071, China; 3 Southwest Jiaotong University emei campus, Emei, 614202, China; 4 Institute of Oncology, Tianjin Union Medicine Centre, Tianjin 300121, China; 5 Optics Research Group, Delft University of Technology, Lorentzweg 1, 2628CJ Delft, The Netherlands

## Abstract

Typically, metal particles are difficult to manipulate with conventional optical vortex (OV) tweezers, because of their strong absorption and scattering. However, it has been shown that the vortex field of surface plasmonic polaritons, called plasmonic vortex (PV), is capable of stable trapping and dynamic rotation of metal particles, especially those of mesoscopic and Mie size. To uncover the different physical mechanisms of OV and PV tweezers, we investigated the force distribution and trapping potential of metal particles. In OV tweezers the stronger scattering force causes a positive potential barrier that repels particles, whereas in PV tweezers the dominant gradient force contributes to a negative potential well, resulting in stably trapped particles. Compared with OV, the orbital angular momentum of PV produces an azimuthal scattering force that rotates the trapped particles with more precise radius and position. Our results demonstrate that PV tweezers are superior in manipulation of metal particles.

An optical vortex (OV) is a beam with helical phase and orbital angular momentum (OAM), and much attention has been paid to its unique properties^1^,^2^. In recent years OV tweezers have been widely used to manipulate microscopic objects, akin to an “optical spanner”. They are effective at both trapping and rotating transparent dielectric particles^3^,^4^,^5^, and as a micromanipulative tool, they show great applicability to micro-fabrication, catalysis, and biomedical science^6^,^7^,^8^.

There are, however, limitations in trapping and manipulating metal particles. The electric charge polarization induces a larger attractive gradient force in metal particles than in dielectric particles, and the repulsive scattering force increases more quickly with increasing particle size than the gradient force does, due to its strong absorptive and scattering properties^9^,^10^,^11^. This means that metal particles in a traditional OV field are more easily pushed away than trapped, especially large metal mesoscopic particles (with radius *a* ~ *λ*) and Mie particles 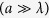. Although several researchers have successfully confined and rotated micrometer metal particles in a ring-shaped OV field, they relied purely on the scattering force^12^,^13^, which resulted in narrow-trapping regions and required high-precision adjustments in the laser beam to maintain the force balance.

Our previous work demonstrated that the whirlpool-like plasmonic vortex (PV) was a counterpart of OV, in terms of evanescent surface plasmonic polaritons (SPPs)^14^. PV was shown be capable of attracting and stably trapping micrometer-sized gold particles, and dynamically rotating them with OAM delivered from an incident OV beam. This implies that PV could be the better candidate for an “optical spanner” of metal objects. There is great potential for applications in catalysis, OAM detection, lab-on-a-chip fabrication and surface-enhanced Raman scattering^15^,^16^,^17^,^18^. However, the physical mechanism by which PV traps and manipulates metal remains a mystery.

Here, we investigate both the plasmonic forces that are exerted on metal particles and the trapping potential in the PV field, to reveal the trapping mechanism and the effect of OAM. To show clearly how the performances of PV and traditional OV tweezers vary, we performed experiments with the same configuration for PV and OV tweezers, but with the metal film removed in the latter as a comparison. The results confirm that in a PV field the metal particles are always attracted and stably trapped, as opposed to being pushed away in the OV field. To explain these observed differences in particle motion, we analyzed the detailed force distributions and trapping potentials of metal particles at different positions in the PV and OV fields. To do this we used the three-dimensional finite difference time domain (FDTD) and Maxwell stress tensor (MST) methods^19^. Through this theoretical analysis, we found that in a PV field, the OAM induced azimuthal scattering force gives rise to particle rotation and overcomes the viscosity resistance. Meanwhile, the radial gradient force produced by the non-uniform field intensity contributes to a negative potential well that stably traps particles. This is more effective than the OV tweezers, which possess a stronger scattering force and a positive potential barrier that repels particles. The advantages of PV tweezers include near-field enhancement of SPPs, dynamic control of particle motion, and a broad applicability to objects of nanometer to micrometer size. We suggest that PV tweezers are a more stable, convenient, precise and general method of trapping and manipulating metal particles.

## Results

### Comparison of PV and OV tweezers experiments

**Figure 1:**
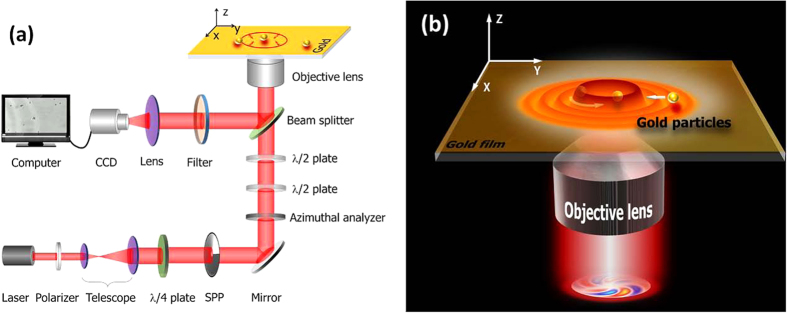
Schematic diagram of experimental setup. (**a**) Optical system used for OV and PV tweezers. A radially polarized optical vortex beam is generated by a quarter-wave plate, a spiral phase plate (SPP) and an azimuthal analyzer and then focused onto the gold film (45-nm thickness). Light wavelength is 1064 nm and the incident power is 100 mW. (**b**) Schematic of metal particles being manipulated with the PV tweezers. The excited plasmonic wave propagates along the surface and is focused to a series of PV rings. The arrows indicate the motion direction of the particles.

Figure 1(a) shows the experimental setup we employed to generate OV and PV tweezers. To compare the behavior of gold particles in both tweezers, the OV and PV experiments were performed in near identical systems. The only difference was that the PV system had a piece of gold film (thickness *d* = 45 nm) covering the glass substrate. The incident light was a near infrared laser with wavelength 1064 nm. The generated OV beam was radially polarized with a topological charge *l* = 5, and it excited SPPs by tightly focusing onto the gold film. The SPPs then propagated toward the field center and interfered in a standing wave pattern similar to a series of concentric donuts, as shown in Fig. 1(b).

**Figure 2:**
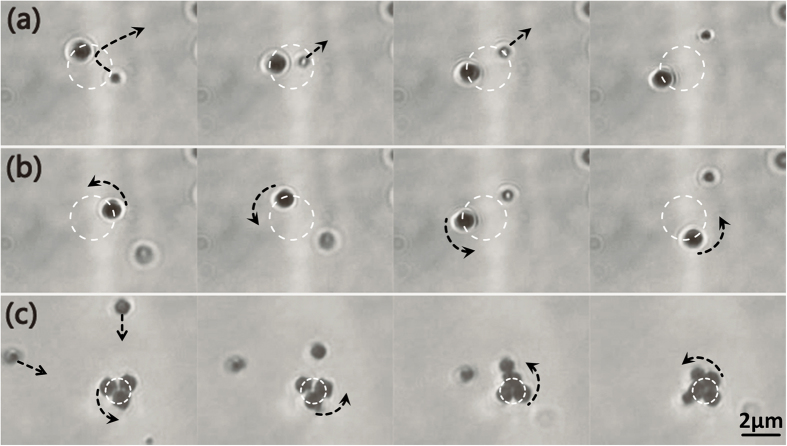
Video recordings of the experiments. (**a**) Repulsive interaction between OV and gold particles. The two particles are the same size but located at different distances from the focal plane. (**b**) Rotation of a particle in the OV field. White-dashed circle in (**a**,**b**) indicates the rotation radius of the particle. (**c**) The particles are captured and rotated by PV. The arrows denote motion direction of particles, and the white circle represents the PV main-lobe donut. Diameter of gold particles is about 1 μm, the working wavelength is 1064 nm, the topological charge of the OV beam is 5, and the incident power is 100 mW.

The experimental results of OV and PV tweezers are shown in Fig. 2 and Supplementary Movies. The OV tweezers were generated by a focused OV beam directly illuminating the glass slide. In that situation it is hard to capture a gold particle by relying on the balance of forces between the particle gravity and the scattering force^12^,^13^. As shown in Fig. 2(a), the gold particle can very easily be bounced out of focus when it approaches the focus donut from the outside. Such a result is due to the vertical scattering force being too strong to balance the gravity force^12^. To solve this issue, the particle needs to be located initially inside the focal donut, then the scattering force will confine the particle to the dark center and rotate it similarly to Rayleigh particles^20^, as shown in Fig. 2(b). As a consequence of this, the OV technique is greatly limited by initial particle position.

In PV tweezers the situation is extremely different. There, the OV beam was focused onto a 45-nm thick gold film where it excited a PV donut field. As shown in Fig. 2(c), the PV tweezers produce stable trapping and rotation of multiple particles inside the PV donut (as demonstrated in our previous work^14^). Additionally, the focused PV donut can even apply its strong gradient force to attract particles from far away, and trap them as well as the plasmonic virtual probe does^19^. This capability removes the limitation on the initial position of particles and makes it more convenient to manipulate metal particles. The rotation radius of particles in the PV donut was a little smaller than in the OV case, because the particles in the PV field were rotating within the focal donut due to the two-dimension nature of SPPs, while the particles in the latter were below the focal plane. The lower rotation speed was due to the wastage during SPPs excitation.

### Force analysis of metal particles in OV and PV fields

To probe the different interactions between particles and the two vortex fields, we numerically investigated the force distributions exerted on metal particles in both OV and PV fields, using FDTD and MST methodology.

**Figure 3:**
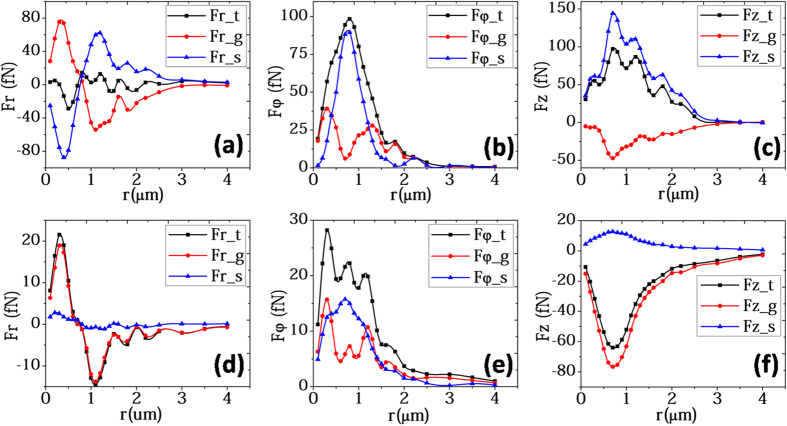
Force distribution analysis in OV and PV fields. The force distributions in (**a**) radial, (**b**) azimuthal and (**c**) *z* direction of a 1-μm-gold particle in the OV field, where *r* represents the distance of particle from the principal optic axis. (**d**–**f**) The same force distributions in the PV field. The gradient force is drawn in red line, the scattering force is in blue line and the resultant force is in black line. The incident power is 100 mW.

In Fig. 3, the upper row presents the force distributions in the radial, azimuthal and *z* directions, respectively, of a gold particle in the focal plane of OV tweezers, during the particle moving outwards from the field center. As shown in Fig. 3(a), the gradient force in the radial direction *F*_*r*_*_g* > 0 when *r* < 0.8 μm, and *F*_*r*_*_g* < 0 when *r* > 0.8 μm. It always points to the high intensity focal donut (at *r* ≈ 0.8 μm) both inside and outside, in accordance with the intensity gradient of the optical field^11^,^21^. The gradient force has two extremes at *r* = 0.4 μm and *r* = 1.1 μm, where the intensity gradient around the focal donut becomes largest. In contrast, the scattering force, *F*_*r*_*_s*, acts in the reverse direction to *F*_*r*_*_g* since it usually originates from the area where high intensity changes to low intensity. *F*_*r*_*_s* is a bit bigger than *F*_*r*_*_g* because of the high focal intensity, so that the resultant force, *F*_*r*_*_t*, impels the particle from the focal donut in the radial direction. The force distribution of particles in the azimuthal direction is depicted in Fig. 3(b), where we observe a peak of the scattering force at the donut region. The strong azimuthal scattering force originates from the helical energy flow of the OV beam^20^ and has the ability to rotate microscopic objects, acting like an “optical spanner”. This is how the OAM of OV works. Meanwhile *F*_*φ*_*_g* is much weaker than *F*_*φ*_*_s* because the OV field is cylindrically symmetrical. In the *z* direction, shown in Fig. 3 (c), the vertical scattering force, *F*_*z*_*_s*, is far larger than the opposing gradient force, *F*_*z*_*_g*, because of the very strong focal intensity of the OV field. Thus the resultant force, *F*_*z*_*_t*, can overcome the gravity force and push the particle out of the focal plane.

The lower row of Fig. 3 shows the force distributions of particles in PV tweezers, in which the particle is placed 50 nm above the gold film, in consideration of the Debye length used in previous reports^19^,^22^. The main differences between the force distributions of OV and PV tweezers are in the radial and *z* directions. Figure 3(d) shows that the radial scattering force, *F*_*r*_*_s*, is extremely small, because the PV field is a SPPs standing wave pattern and has an almost zero power flux. This means that the gradient force plays a dominant role and attracts particles in the radial direction to the central donut, leading to stable trapping. As the area of PV field can be dynamic adjust by changing the distance between metal film and object lens, so the actual area of PV field might be slightly larger than simulations, and combine with the effect of heating, the actuating range of trapping force could be a bit larger in experiment. The force distribution in the azimuthal direction, shown in Fig. 3(e), is similar to that in Fig. 3(b), which implies that the PV still retains the OAM of the incident OV even after SPPs excitation. As the evanescent SPPs decay very quickly in the *z* direction^23^, the vertical scattering force, *F*_*z*_*_s*, is much smaller than the gradient force, *F*_*z*_*_g*, as shown in Fig. 3(f). This leads to a strong resultant attractive force pointing towards the gold film, drawing the particle to the bottom film in the PV field, as opposed to being pushed out of focus as in the OV tweezers.

### Trapping potential of particles in OV and PV fields

**Figure 4:**
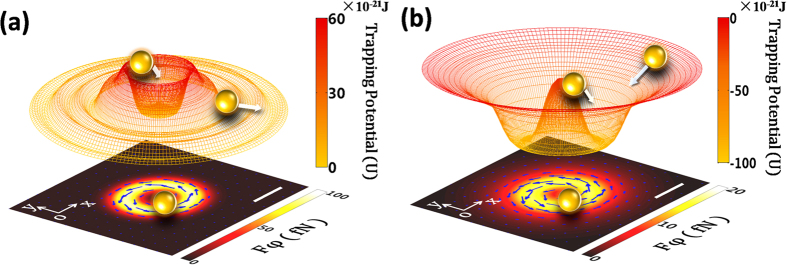
Trapping potential of OV and PV for metal particles. Trapping potential of a 1 μm gold particle in OV (**a**) and PV (**b**) fields, respectively. The particle is pushed away in the OV field, but drawn and captured in the PV field, where it is located close to the focal donut. The images below show the azimuthal force exerted on the particle in the focal plane. The arrows represent the direction of the force. The length of scale bar is 1 μm.

To clearly visualize the mechanisms for the optical trap, we calculated the trapping potential for both vortex fields. The details of the potential calculations are described in the Methods section. The potential well shown in the upper diagrams of Fig. 4 describes the radial optical force, *F*_*r*_*_t*, exerted on the particle. We can clearly see the different behaviors of gold particles in OV and PV fields. For OV fields, the stronger radial scattering force (Fig. 3(a)), means that the potential, *U*, creates a positive potential barrier at the focal donut, just like a round wall isolated from its surroundings. This will exclude any particles that approach the donut from the outside, and confine the particles inside (whose initial positions are in the focal donut). This OV tweezers trapping mechanism has been confirmed by many previous OV trapping studies^12^,^13^,^22^. Here, the peak value of *U* is |*U*_*max*_| = 62 × 10^−21^ J, at a position *r* = 0.7 μm, normalized by 100 mW of the incident power, the height of the potential barrier is calculated to be 14.8 *k*_*B*_*T*, with a system temperature *T* = 300 K. It is hard for particles to break through this barrier. The bottom figure of Fig. 4(a) shows the azimuthal optical force exerted on a particle by the OAM of OV, where the arrows indicate the direction of the force.

In contrast to the OV tweezers, the PV tweezers possess an attractive gradient force that dominates in the radial direction (Fig. 3(d)). Thus, in Fig. 4(b) the trapping potential, *U*, presents a negative potential well at the donut, causing a particle to drop to the bottom of the potential well regardless of its position, inside or outside. The normalized peak potential |*U*_*max*_| *=* 94 × 10^−21^ J is obtained at a position *r* = 0.8 μm. The depth of the PV potential well obtained by the simulation is 22.7 *k*_*B*_*T* per 100 mW incident power, and the stability number *S* = 22.7 (defined in the Methods section). These satisfy the requirements of stable optical trapping^24^. The bottom figure of Fig. 4(b) shows the azimuthal force caused by the OAM of PV. This has the potential to rotate particles as effectively as OV tweezers, and to verify that the OAM of the incident OV beam has been transferred to the PV successfully. The trajectory and velocity of particle motion in the PV field could be controlled by polarization, wavelength and topological charge of the incident OV beam, providing a good possibility of achieving precise particle manipulation and OAM detection^14^.

### Effects of particle size and the OV topological charge

**Figure 5:**
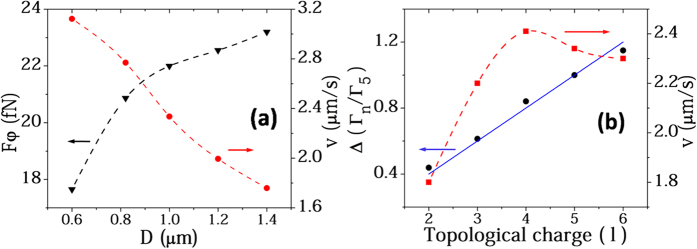
Influences of particle size and topological charge on the force. (**a**) The azimuthal force exerted on gold particles with different sizes (black line) and the corresponding rotational speed (red line) at the peak of the PV donut. (**b**) *Г*_*n*_ represents the torque of a particle in the PV with a topological charge of *l* *=* *n*, and *Δ* is the ratio of *Г*_*n*_ to *Г*_*5*_. Blue line and black dots show the theoretical prediction and FDTD result of *Δ*, respectively. Red line shows the corresponding rotational speed of the particle in a different PV field. All results are normalized by 100-mW-incident power.

Our previous experimental results^14^ demonstrated that the radius and speed of particle rotation in a PV field is influenced by the topological charge of the incident OV beam. To further investigate the rotational dynamics, we numerically probed the effects of gold particle diameter (0.6~1.4 μm) and OV topological charge (2~6) on the optical forces and particle movement. The calculation details are described in the Methods section. In Fig. 5(a), it can be seen that the azimuthal force provided by OAM increases with the particle diameter, because of force area amplification. Generally the azimuthal optical force is offset by the drag force in solution as *F*_*φ*_ = *F*_drag_ = *6πηav* at a stable rotation speed *v*, where *v* is proportional to the ratio between *F*_*φ*_ and particle radius, *a*. In Fig. 5(a) the speed, *v*, decreases with particle size because the increase of *F*_*φ*_ is slower than the increase of *a*.

Figure 5(b) shows the torque, *Г*_*n*_, and speed, *v*, of a particle at the donut peak of the PV field as a function of topological charge, *l*, from 2 to 6. The torque, *Г*_*n*_, at *l* = n can be expressed as *Г*_*n*_ *=* *v*·*R*_*n*_, where *R*_*n*_ is the radius of particle rotation. According to previous research^25^,^26^, *Г* is directly proportional to *l* (see Methods section), so the rotational speed, *v*, increases with the topological charge. It reaches a peak value at *l* = 4 then declines at larger *l* because the larger radius of the donut eventually reduces the average intensity of the donut. When changing the topological charge of the incident OV beam, the torque is expected to follow the proportional relation *Г*_*n*_/*Г*_*m*_ = *n/m*^26^,^27^. We defined the ratio of torques between *l* = n and *l* = 5 as *Δ* = *Г*_*n*_/*Г*_*5*_, and show the theoretical result from the proportional relation and the FDTD in Fig. 5(b). Both calculations of *Δ* are directly proportional to the topological charge and thus agree well. The slight difference could be caused by the heat wastage during SPPs excitation^28^,^29^,^30^,^31^. Here, the ratio *Г*_*5*_/*Г*_*2*_ we calculate is about 2.28, which is similar to the previous experimental result^14^.

### The heating effect in the trapping of PV tweezers

**Figure 6:**
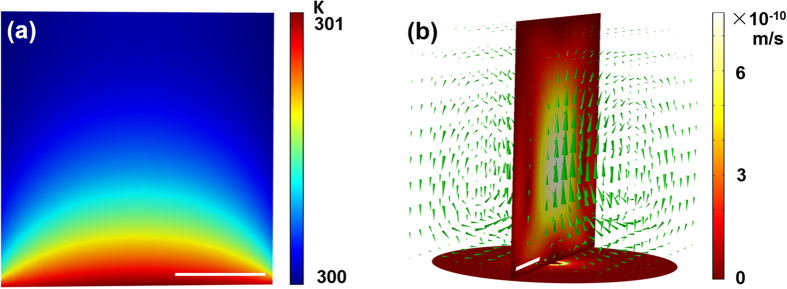
Temperature distribution and thermal convection in PV tweezers. (**a**) Temperature distribution on gold surface caused by the exciting of SPPs, the bottom is the gold film surface; (**b**) Velocity of thermal convection in PV tweezers. The circinal figure below is the excited PV field, and the perpendicular section indicates the velocity distribution of convection. The green arrows demonstrate the directions and magnitudes of thermal convection, the color bar indicates the velocity. White scale bar in both figures is 5 μm.

In addition to the optical forces studied above, we noted that heating (temperature changing and thermal convection) also influences some plasmonic trapping systems^32^. The maximum incident power we used in experiment is 100 mW, so we processed a series of thermal simulations with 100 mW incident power to calculate the temperature distribution and thermal convection of PV tweezers. The results are shown in Fig. 6. The parameters used in simulations are corresponding with experiment, and the environmental temperature is set as 300 K.

Figure 6(a) shows the temperature distribution, which is caused by Joule effect of SPPs over a large area of gold film. Due to the high thermal conductivity of gold film 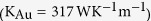, thermal energy conducts to the whole gold film in microseconds. Therefore, the highest temperature increment at the film is only about 1.1 Kelvin. Figure 6(b) shows the thermal convection caused by photothermal effect. The thermal convection works in the range of the entire volume of water and drive particles to the center of PV field, which might contribute to the capture of PV tweezers. Speed of convection is labeled by the size of arrows and color bar, which is of sub-nanometer per second order, thus the corresponding drag force is quite small compared with the optical force. As a result, the influence of heating effect is quite a few in such plasmonic tweezers, similar to our previous study^19^,^33^.

## Discussion

In this paper, we aimed to reveal the different mechanisms of metal particle trapping and rotation in OV and PV fields, and to prove the superiority of our novel PV tweezers in trapping and manipulating metal particles. Our experimental results in Fig. 2 clearly demonstrate that although OV tweezers can rotate confined gold particles, they have difficulties in capturing particles from outside the donut, whereas PV tweezers have the ability to trap particles from far away (~10 λ) and rotate them.

Our theoretical calculations and force analysis verify our experimental results. It is well known that for metallic particles, the attractive gradient force is essentially the Coulomb force induced by electric charge polarization of metal particles, and that the repulsive scattering force is due to the radiation pressure induced by the high reflection and absorption of the metal. In OV tweezers, the stronger scattering force at the focal donut region contributes to a positive potential barrier (Fig. 4(a)) that is hard for the particles to climb over and become trapped within the donut. In PV tweezers, the dominant gradient force at the donut region forms a negative potential well (Fig. 4(b)), which is deep enough to stably trap metal particles from a long distance. The azimuthal scattering force produced by OAM can overcome the damping force and rotate particles in both tweezers. It shows a marvelous harmony in the process of PV rotating gold particle: the radial gradient force in the horizontal *x-y* plane provides the trapping and centripetal force whilst the azimuthal scattering force overcomes the viscosity resistance and rotates the particle, and the gradient force in the *z* direction confines the particle close to the metal film. The three forces work together to produce stable particle motion. The influence of particle size and topological charge of optical forces were also studied (Fig. 5) and agree well with both theoretical predictions^26^ and experimental results^14^.

In conclusion, we experimentally demonstrated the capture and rotation of micrometer-diameter gold particles by PV tweezers, and theoretically analyzed the force distribution and trapping potential to reveal the physical mechanism of the PV. We suggest that PV tweezers are superior in manipulating metallic objects when compared with the performance of traditional OV tweezers. The experimental and theoretical results confirm that the OAM of the incident OV beam can be transferred to the PV field, along with its dynamic properties. We further studied the effects of metal particle size and the topological charge of the incident OV beam on the manipulation capabilities of PV tweezers. And heating effect to the trap of PV tweezers is particularly simulated and analyzed. We found that PV tweezers could be a more universal and stable approach to the manipulation of metallic objects, with possible applications in catalysis, OAM detection, micro-fabrication, lab-on-a-chip device and other related fields. We believe that this work is not only a step towards a more competitive method of manipulating metallic objects, but that it also contributes to a deeper understanding of the dynamic characteristics of SPPs fields.

## Methods

### Generation of PV tweezers

The optical system we used is shown in Fig. 1(a). The laser source was a near-infrared laser (100 mW, 1064 nm), which created a circularly polarized OV with topological charge *l* = 5, after passing through a polarizer, quarter-wave plate and spiral phase plate with topological charge *l* = 4^14^. To transform the polarization state, an azimuthal-type polarization analyzer and two half-wave plates at 45° to each other (acting as polarization rotators^34^) were used to produce a radially polarized final OV beam with charge *l* = 5. The OV beam was focused through an oil immersion objective (100 X, NA = 1.49) onto a glass slide (*n*_1_ = 1.515) covered with a 45-nm-thin-gold film (*n*_2_ = 0.272 + 7.07i at a wavelength of 1064 nm). The SPPs were excited at the surface plasmon resonance angle on the top surface of gold film then propagated towards the center where they interfered into a PV circle^35^, as Fig. 1(b) shows. The reflected light from the particles propagated back through the lens and was finally imaged by a CCD camera. A 1000-nm lowpass filter was placed in front of the camera to eliminate the influence of incident light. The gold particle samples, with diameter about 1 μm, were diluted by deionized water.

### Calculations of force, trapping potential and torque

We performed a series of three-dimension FDTD simulations for OV and PV tweezers to investigate the interaction between metal particles and the PV field. All simulation parameters were chosen to match experimental conditions. The FDTD simulations provided the distributions of all electromagnetic field components around the particle, and then we imputed these electromagnetic field distributions into the MST to obtain the optical force exerted on the particle. Following the MST theory, the time-average force of mesoscopic particles can be expressed as


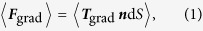



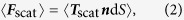


in which ***T***_grad_ and ***T***_scat_ represent the tensor of the gradient force and scattering force, respectively, d*S* is the integral area and ***n*** is the unit normal perpendicular to it^19^.

According to these equations, a detailed force distribution of particles in the light field can be calculated from the electromagnetic field distribution obtained from the three dimensional monitor surrounding the particle. The total force on the metal particle from the optical field can be divided into the gradient force and the scattering force in good approximation^19^.

The trapping potential in each optical field determines the stability of the trap, and can be obtained from the force distribution in the radial direction by the expression


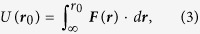


where *U(**r***_*0*_) is the required energy to move the particle from the trap to infinity^36^. To measure the stability of the trap, we introduced the stability number, *S*


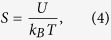


where *k*_*B*_ is the Boltzmann constant, and *T* is the temperature^24^. *k*_*B*_*T* is used to represent the random thermal motion of the particle. In principle, an *S* = 1 potential well is sufficient to overcome the thermal motion of a particle and confine it in a trap^24^, but it has been proposed that an *S* = 10 potential well would be required for a stable optical trapping^6^.

The damping force exerted on the particle in the experiment can be expressed by the function *F*_drag_ = 6π*ηav*, where *η* and *a* represent the coefficient of viscosity of water and the radius of particle, respectively. *v* is the rotational speed of the particle, and can be extracted from the CCD videos. We obtained the azimuthal optical force from the relation 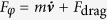
15,16,19, in which 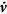 is the acceleration of particle. Generally, we considered the particle to be moving in a uniform circular motion.

According to the theory developed by Allen^26^,^27^, the torque of a particle is


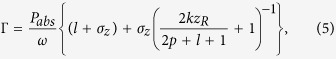


where 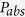 is the absorption power of the particle, *ω* is the frequency of light, *p* is the mode indices, *k* is the wave number and and *z*_*R*_ is a length term, *σ*_*z*_ is ±1 for circularly light and 0 for plane-polarized light. The ratio of the torque of the particle in PV with different topological charge will be *Г*_*n*_/*Г*_*m*_ = *n/m*, in which *n* and *m* is the value of topological charge of the vortex, and *Г*_*n*_ can be expressed as *Г*_*n*_ *=* *v*·*R*_*n*_, where *R*_*n*_ is the radius of the rotation. We also defined the ratio *Δ* = *Г*_*n*_/*Г*_5_ to compare the calculated results with the theoretical predictions^26^ and experimental results^14^.

## Additional Information

**How to cite this article**: Zhang, Y. *et al*. A Plasmonic Spanner for Metal Particle Manipulation. *Sci. Rep*. **5**, 15446; doi: 10.1038/srep15446 (2015).

## Supplementary Material

Supplementary Information

Supplementary Movie s1

Supplementary Movie s2
