# Supplementary material for: A Plasmonic Spanner for Metal Particle Manipulation
Source: Sci Rep. 2015 Oct 20;5:15446. doi: 10.1038/srep15446 (PMC4612544; doi:10.1038/srep15446)
Supplement: Supplementary Information [file srep15446-s1.doc]

**A Plasmonic Spanner for** **Metal Particle Manipulation**

Yuquan Zhang^1*^, Wei Shi^2*^, Zhe Shen^2^, Zhongsheng Man^2^, Changjun Min^1#^, Junfeng Shen^3^, Siwei Zhu^4^, H. Paul Urbach^5^ and Xiaocong Yuan^1#^

^1^ Institute of Micro and Nano Optics, Key Laboratory of Optoelectronic Devices and Systems of Ministry of Education and Guangdong Province, College of Optoelectronic Engineering, Shenzhen University, Shenzhen, 518060, China

^2^ Institute of Modern Optics, Nankai University, Tianjin, 300071, China

^3^ Southwest Jiaotong University emei campus, Emei, 614202, China

^4^ Institute of Oncology, Tianjin Union Medicine Centre, Tianjin 300121, China

^5^ Optics Research Group, Delft University of Technology, Lorentzweg 1, 2628CJ Delft, The Netherlands.

* These authors contributed equally to this work.

^#^ Correspondence and requests for materials should be addressed to C.M. (email: [cjmin@szu.edu.cn](mailto:cjmin@szu.edu.cn)) and X.Y. (email: [xcyuan@szu.edu.cn](mailto:xcyuan@szu.edu.cn)).

**Legends for Supplementary Movies**

Supplementary Movie 1:

Experimental results of gold particles motion in OV tweezers, where gold particle can very easily be bounced out of focus when it approaches the focus donut from the outside due to the strong scattering force. Diameter of gold particles is about 1 μm, the wavelength of trapping laser is 1064 nm, the topological charge of the OV beam is 5, and the incident power is 100 mW.

Supplementary Movie 2:

Experimental results of gold particles motion in PV tweezers. PV tweezers produce stable trapping and rotation of multiple particles inside the PV donut near to the film. Diameter of gold particles is about 1 μm, the wavelength of exciting laser is 1064 nm, the topological charge of the OV beam is 5, and the incident power is 100 mW.
